# Fear Processing in Dental Phobia during Crossmodal Symptom Provocation: An fMRI Study

**DOI:** 10.1155/2014/196353

**Published:** 2014-03-11

**Authors:** Kevin Hilbert, Ricarda Evens, Nina Isabel Maslowski, Hans-Ulrich Wittchen, Ulrike Lueken

**Affiliations:** ^1^Institute of Clinical Psychology and Psychotherapy, Technische Universität Dresden, Chemnitzer Straße 46, 01187 Dresden, Germany; ^2^Neuroimaging Center, Technische Universität Dresden, Chemnitzer Straße 46a, 01187 Dresden, Germany

## Abstract

While previous studies successfully identified the core neural substrates of the animal subtype of specific phobia, only few and inconsistent research is available for dental phobia. These findings might partly relate to the fact that, typically, visual stimuli were employed. The current study aimed to investigate the influence of stimulus modality on neural fear processing in dental phobia. Thirteen dental phobics (DP) and thirteen healthy controls (HC) attended a block-design functional magnetic resonance imaging (fMRI) symptom provocation paradigm encompassing both visual and auditory stimuli. Drill sounds and matched neutral sinus tones served as auditory stimuli and dentist scenes and matched neutral videos as visual stimuli. Group comparisons showed increased activation in the insula, anterior cingulate cortex, orbitofrontal cortex, and thalamus in DP compared to HC during auditory but not visual stimulation. On the contrary, no differential autonomic reactions were observed in DP. Present results are largely comparable to brain areas identified in animal phobia, but also point towards a potential downregulation of autonomic outflow by neural fear circuits in this disorder. Findings enlarge our knowledge about neural correlates of dental phobia and may help to understand the neural underpinnings of the clinical and physiological characteristics of the disorder.

## 1. Introduction

Specific phobia is the most prevalent anxiety disorder and among the most common mental disorders in general [1, 2]. According to DSM IV-TR and DSM-5 criteria, specific phobia is characterized by marked and unreasonable fear towards a specific object or situation which is almost always provoked whenever the phobic stimulus is not avoided [3, 4]. In the last decade, an increasing number of studies investigated the neural substrates of specific phobia, identifying mainly the amygdala, insula, and anterior cingulate cortex (ACC) as core components of the underlying neural network involved in the processing of threat [5, 6]. However, while these results have been proven to be consistent and stable, they are almost exclusively based upon studies investigating the animal subtype of specific phobia, most notably spider phobia. Literature on the other subtypes—blood-injection-injury (BII), situational, natural environment, and other specific phobia—is rare and focuses mostly on BII phobia, which includes dental phobia [7]. Unfortunately, results are more inconsistent here: while some studies reported increased activation in the insula or ACC as well [8, 9], others mainly found differential activation compared to healthy controls in orbitofrontal and prefrontal areas [9–11]. Results also yielded patterns of activation in other areas such as the thalamus [12] or were not indicative of any significant difference between groups in any area [13, 14]. No study so far replicated the finding of amygdala hyperactivation as repeatedly reported for animal phobia.

These diverging findings regarding the notable lack of activation in cortical and subcortical structures involved in the processing of threat were subject to different interpretations. Among others, a dissociation of subjective and physiological fear reactions [14] or altered cognitive control or emotional regulation processes [10, 11] have been proposed. However, methodological causes are possible as well. As pointed out by Köchel et al. [15], fMRI studies to date have used visual stimuli to induce anxiety without exception. In dental phobics, however, visual stimuli are often less anxiety inducing than stimuli using other sensations [16], which could confound results from studies in BII phobia that often include dental phobics as well [9, 10, 13, 14]. Therefore, inconsistent findings in BII phobia might partly result from the use of stimuli that do not maximally trigger dental fears when investigating group differences in brain activation patterns.

We therefore aimed to further elucidate the influence of stimulus modality on neural fear processing in dental phobics (DP). DP and healthy controls (HC) underwent a symptom provocation paradigm using both auditory and visual stimuli. Autonomic markers (skin conductance) were recorded online. We expected DP not only to show enhanced subjective anxiety towards dental stimuli in general when compared to controls but also to react specifically stronger towards the auditory than visual stimuli. Moreover, we expected stronger autonomic arousal particularly in response to auditory stimuli. On the neural level, we hypothesized DP to show increased brain activation in the amygdala, ACC, insula, thalamus, and orbitofrontal cortex (OFC) compared to HC, particularly during auditory symptom provocation but not during visual phobic stimuli. Based on the finding of a positive relationship between the level of activation in these areas and symptom severity as reflected by subjective and autonomic markers [13, 14], we also expected such a correlation to be present in the current study.

## 2. Methods

### 2.1. Subjects

Thirteen dental phobics (DP) and thirteen healthy controls (HC) were recruited from a pool of participants from an online screening. Inclusion criteria were a sum score of 72 or above in the Dental Fear Survey (DFS; indicating moderate to severe dental phobia; [17]) for DP and a sum score of 33 or below (being a score in the lower quartiles) for the HC. Exclusion criteria were fMRI-related exclusion criteria, psychotropic medication less than four weeks prior to assessment, any lifetime neurological disease, or the following current mental disorders (12-month prevalence): bipolar disorder, psychotic disorder, posttraumatic stress disorder, substance dependence, severe major depressive disorder, and comorbid animal type of specific phobia. Psychiatric diagnoses were determined by the Composite International Diagnostic Interview (CIDI; [18]) for DSM IV-TR and confirmed by clinical experts. In total, 2 dental phobics had one comorbid disorder (*n* = 1 panic disorder with agoraphobia, *n* = 1 alcohol abuse) and 4 dental phobics had at least two comorbid disorders (*n* = 1 panic disorder with agoraphobia, *n* = 1 panic disorder without agoraphobia, *n* = 1 agoraphobia without history of panic disorder, *n* = 2 social anxiety disorder, *n* = 1 specific phobia “other” subtype, *n* = 1 eating disorder, *n* = 2 obsessive compulsive disorder, *n* = 1 dysthymia, *n* = 1 conversion disorder, and *n* = 1 dissociative disorder not otherwise specified). HC were free of any DSM IV-TR diagnoses. Additionally, the sample was characterized via questionnaires on depressiveness [19], anxiety sensitivity [20], and broadly defined symptom severity of BII phobia [21]. The study protocol was approved by the ethics committee of the Technische Universität Dresden.

### 2.2. Experimental Procedure

An fMRI block-design symptom provocation task applying audio and video stimulus materials was programmed on Presentation 12.0 (Neurobehavioral Systems, Albany, CA, USA) software and presented using video goggles (VisuaStim Digital, Northridge, CA, USA). Auditory stimuli comprised a set of 10 dental drill sounds available from a commercial website (http://www.audiosparx.com/) and 10 neutral sinus tone stimuli in different frequencies that were custom made. Sufficient volume was used such that sounds were well discriminated above scanner noise but not uncomfortable for subjects. Visual stimuli comprised a set of 10 previously validated videos [14, 22], depicting anxiety arousing dentist scenes and 10 neutral stimuli matched for information complexity, movements, timing, and background textures. Stimuli were presented for 15 seconds each, separated by a jittered inter-stimulus-interval ranging between 11 to 19 seconds. Thus, there were four conditions in total: dental audio neutral (DAN), dental audio anxiety (DAA), dental video neutral (DVN), and dental video anxiety (DVA). These conditions were presented in pseudorandomized order with no conditions being presented more than two times in a row. Following the fMRI paradigm, all subjects rated all stimuli offline for valence “the picture was negative/neutral/positive,” arousal “the picture made me nervous: not at all/very,” anxiety “the picture made me anxious: not at all/very,” disgust “the picture was disgusting: not at all/very,” and pain “the picture made me feel/remember pain: not at all/very” on nine-point Likert scales, similar to earlier studies [13, 14]. During rating, all stimuli were presented in pseudorandomized order as well. Subjective ratings from *n* = 2 subjects (*n* = 1 DP, *n* = 1 HC) were incomplete and therefore excluded from the analysis.

SC responses were recorded online using Ag/AgCl electrodes (MES Medizintechnik, Munich, Germany) affixed to the second phalanx of the nondominant hand's index and middle finger, with isotonic electrode paste (Synapse, Kustomer Kinetics, Arcadia, CA, USA) as contact medium and Brain Vision ExG Amplifier and Brain Vision Recorder (Brain Products, Munich, Germany) as hard- and software. An initial sampling rate of 1000 Hz and 10 sec high-pass and 250 Hz low-pass filters were used with a response criterion of 0.02 *μ*S. The Matlab-based application Ledalab Version 3.4.3 [23, 24] was used for SC data processing, during which the sampling rate was changed to 10 Hz. A continuous decomposition analysis was applied to the data to extract the phasic driver (CDA.SCR) and tonic (CDA.tonic) SC activity within the 1–15 sec time window after stimulus onset. Data were range corrected according to Lykken [25]. SC data from *n* = 2 subjects (DP) were lost due to technical failure.

### 2.3. Analysis of Demographic, Behavioral, and Physiological Data

Chi-square tests and independent *t*-tests were applied to demographic and questionnaire data as appropriate. Ratings were compared between groups for each dimension by repeated-measurement analyses of variance (ANOVAs), with the between-subject factor group (DP; HC) and the within-subject factors stimuli (audio; video) and condition (anxiety; neutral). Testing for normal distribution of SC parameters using Shapiro-Wilk tests indicated a nonnormal distribution of SC data. Therefore, SC data was log transformed at first and then analyzed by repeated-measurement ANOVAs with the between-subject factor group (DP; HC) and the within-subject factors stimuli (audio; video) and condition (anxiety; neutral), for tonic and phasic SC components separately. Pairwise comparisons were employed as post hoc tests. SPSS 20 was used for all analyses, with the level of significance being set at *P* < 0.05.

### 2.4. fMRI Data Acquisition and Analysis

A 3-Tesla Trio-Tim MRI whole-body scanner (Siemens, Erlangen, Germany) and a 12 channel head coil were used for MRI data collection. Functional images were acquired via T2∗ weighted gradient echo planar imaging (EPI) covering the whole brain (560 volumes, repetition time (TR) 2500 msec, echo time (TE) 25 msec, field of view 192 × 192 mm, and matrix 64 × 64). 44 axial slices were recorded in tilted angle (AC-PC + 30°; interleaved acquisition, no gap, slice thickness 3 mm, and in-plane resolution 3 × 3 mm) to reduce susceptibility artifacts in inferior brain areas [26]. Four dummy volumes were discarded with regard to T1 equilibration effects. The T1 weighted structural reference image was acquired via Magnetization Prepared Rapid Gradient Echo Imaging (MPRAGE; 176 sagittal slices, slice thickness = 1 mm, TE = 2.26 msec, TR = 1900 msec, flip angle = 9°, FOV = 256 × 256 mm^3^, and matrix = 256 × 256). Headphones were applied for stimulus presentation, as hearing protection, and to allow communication with the subject. fMRI data were analyzed using SPM8 (Wellcome Trust Centre for Neuroimaging, UCL, London, UK). Images were realigned and unwarped to correct for head movement, applying a fieldmap correction to the EPI time series. Structural and functional images were coregistered, segmented, and normalized to the MNI reference brain (Montreal Neurological Institute, Quebec, Canada). Functional data was upsampled to 2 × 2 × 2 mm voxel size. An 8 mm full-width half-maximum Gaussian kernel was applied for spatial smoothing.

On subject level, four regressors of interest (DAN > BL, DAA > BL, DVN > BL, and DVA > BL) and the six-movement parameters as regressors of no interest were introduced to the general linear model. Results were included in a flexible factorial model for a random effects analysis on group level. The subjects factor, group factor (HC, DP), and stimulus factor (DAN > BL, DAA > BL, DVN > BL, and DVA > BL) were specified and an additional interaction between group and stimulus factors was modeled. The following contrasts were tested: auditory (DAA > DAN) or visual (DVA > DVN) stimulus material, between groups and auditory versus visual stimulus material ((DAA > DAN) > (DVA > DVN)) and vice versa, between groups. As differences between two modalities of the same phobic material might not be of large effect size, a Monte Carlo simulation was used to determine a cluster size-based significance threshold [27]. This approach has been shown to be more sensitive to small effects than the standard 0.05* familywise error* (FWE) correction, while still being an adequate correction for multiple comparisons [28]. The cluster size was calculated by assuming an individual voxel type I error of *P* < 0.001 and including the study's matrix, slice number, smoothing kernel, and (upsampled) voxel size. 10000 iterations determined a minimum cluster size of 58 consecutive voxels. Since no study investigated the neural correlates of auditory symptom provocation in DP before, an exploratory whole brain analysis was employed. Additionally, a region-of-interest analysis (ROI) was conducted for the amygdala, as the cluster-based significance threshold used here might require too many consecutive voxels for such a rather small structure. Estimated beta values of the insula and OFC were extracted clusterwise via the first eigenvariate and correlated with DFS sum scores and tonic and phasic SCRs towards auditory symptom provocation within the DP. Other estimated beta values were extracted accordingly for illustration.

## 3. Results

### 3.1. Sample Characteristics and Behavioral Data

Sample characteristics and clinical data are presented in Table 1. DP rated both auditory and visual stimulus material more negatively than HC (main effect group: valence: *F*(1, 22) = 13.514, *P* < 0.01; arousal: *F*(1, 22) = 15.643, *P* < 0.001; anxiety: *F*(1, 22) = 40.550, *P* < 0.001; disgust: *F*(1, 22) = 14.273, *P* < 0.01; pain: *F*(1, 22) = 43.769, *P* < 0.001). A main effect of stimulus material was detected for pain and valence (valence: *F*(1, 22) = 24.324, *P* < 0.001; pain: *F*(1, 22) = 6.020, *P* < 0.05; all other dimensions above *P* > 0.07), indicating that auditory stimulus material was rated as partially more negative than visual stimulus material. However, this finding was not driven particularly by one of both groups (interaction effect: group ∗ stimulus material: all interactions above *P* > 0.07). Anxiety arousing stimuli were rated as more negative than neutral stimuli (main effect condition: valence: *F*(1, 22) = 109.427, *P* < 0.001; arousal: *F*(1, 22) = 59.586, *P* < 0.001; anxiety: *F*(1, 22) = 56.854, *P* < 0.001; disgust: *F*(1, 22) = 40.195, *P* < 0.001; pain: *F*(1, 22) = 69.147, *P* < 0.001). Post hoc analyses on the group ∗ condition interaction (valence: *F*(1, 22) = 17.040, *P* < 0.001; arousal: *F*(1, 22) = 31.930, *P* < 0.001; anxiety: *F*(1, 22) = 33.384, *P* < 0.001; disgust: *F*(1, 22) = 18.146, *P* < 0.001; pain: *F*(1, 22) = 24.458, *P* < 0.001) indicated that significant group differences were present for anxiety arousing stimuli in all dimensions (all below *P* < 0.001); however, group differences emerged for anxiety and pain dimensions towards neutral stimuli as well (anxiety: *P* < 0.05; pain: *P* < 0.05; all other dimensions above *P* > 0.17). The three-way interaction was not significant (interaction effect: group ∗ stimulus material ∗ condition: all dimensions above *P* > 0.05).

**Figure 1:**
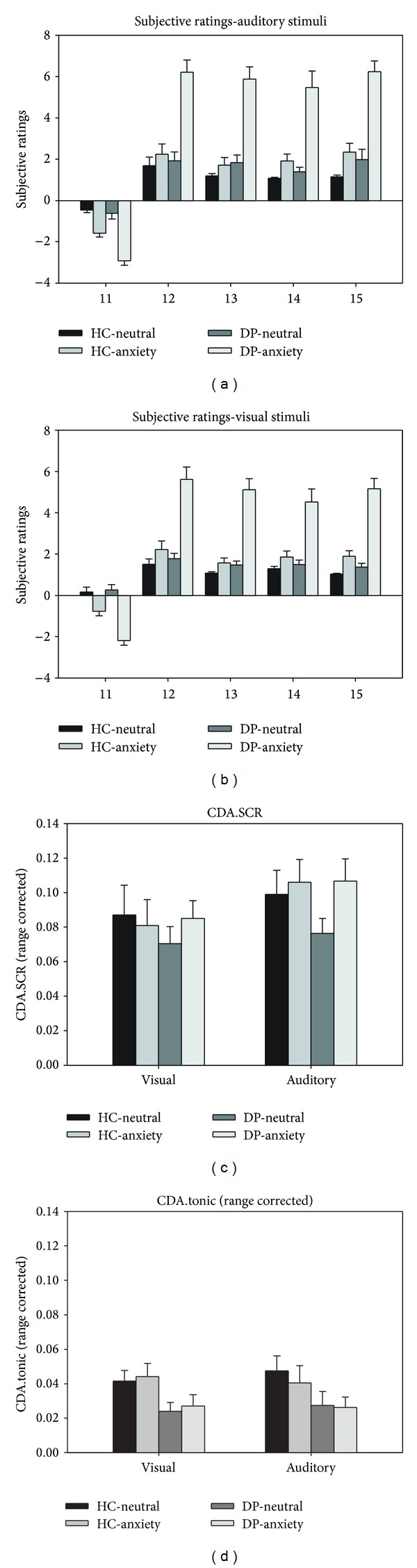
Behavioral data. Upper half: subjective ratings for auditory (a) and visual (b) dental stimuli. Lower half: phasic (CDA.SCR; (c)) and tonic (CDA.tonic; (d)) skin conductance responses. HC: healthy control group; DP: dental phobia group. *P < 0.05; **P < 0.01; ***P < 0.001.

Regarding the physiological data, there was a main effect of stimulus material for CDA.SCR (*F*(1, 22) = 5.880, *P* < 0.05), indicating higher CDA.SCR towards auditory than visual stimuli. No other significant main effects or interactions emerged (all above *P* > 0.05). Regarding CDA.tonic, there was a nonsignificant trend towards the main effect of group (*F*(1, 22) = 3.028, *P* = 0.096) hinting on a slightly higher tonic SC level in the HC. Beside this trend, no main effects or interactions showed true significance (all above *P* > 0.17). Subjective ratings and physiological data are depicted in Figure 1.

### 3.2. fMRI Results

**Figure 2:**
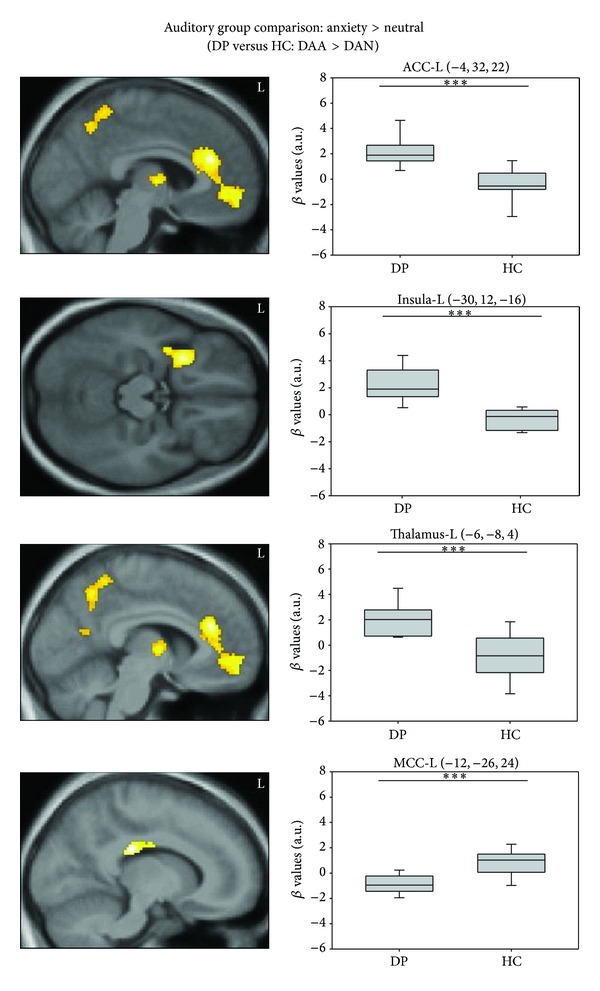
Neural activation patterns of the between-group comparison for auditory stimulus material (DAA > DAN): dental phobics versus healthy controls (upper three) and healthy controls versus dental phobics (below). DP: dental phobia group; HC: healthy control group; ACC: anterior cingulate cortex; MCC: middle cingulate cortex; L: left side; analysis: minimum cluster size = 58; *P < 0.05; **P < 0.01; ***P < 0.001.

Table 2 gives a summary of the whole-brain findings in all contrasts. Direct group comparisons for the auditory stimulus material resulted in significantly increased activation in the ACC, insula, thalamus, inferior frontal gyrus, hippocampus, precuneus, postcentral gyrus, and calcarine sulcus in DP but only in the MCC for HC (see Figure 2). During visual stimulation, considerably less differential brain activation was found, with increased activation in the vermis in the DP being the only significant difference. When finally comparing neural activation during auditory versus visual stimulation between groups, DP showed increased activation in the insula, OFC, and precuneus for auditory versus visual stimuli and in the caudate nucleus for visual versus auditory stimuli. For all contrasts, the ROI approach yielded no additional amygdala activation.

Results of the correlational analyses can be inspected in Table 3. A significant negative correlation emerged between OFC activation during visual stimulation and corresponding CDA.tonic. No other correlations were significant; however, two nonsignificant trends were observed for correlations between insula activation during auditory stimulation and CDA.tonic and between insula activation during visual stimulation and CDA.phasic. Again, a negative correlation was indicated.

## 4. Discussion

This study investigated the effect of crossmodal phobic stimulus processing on neural correlates in dental phobia. The following main findings were observed: (1) while both auditory and visual dental anxiety stimuli were rated as more aversive from DP versus HC, (2) DP showed increased neural activation under auditory dental anxiety stimuli only in most areas related to phobic fear in the animal subtype (except the amygdala). (3) Despite this activation in neural substrates indicative of threat processing, no differential activation was observed in autonomic arousal markers. Negative correlations between neural and autonomic markers could indicate downregulation of autonomic reactions.

The symptom provocation paradigm applied in this study made use of research on the hierarchy of feared situations in dental phobia [16] and included auditory stimuli in order to find a more powerful and robust trigger for phobic fears in these samples. Subjective ratings confirmed that symptom provocation was successful with both visual and auditory stimulus materials. However, subjective ratings also indicate that auditory and visual stimulus materials differed regarding their pain-inducing quality and subsequent overall valence, with auditory stimuli being more painful and aversive. These results are in line with pain being proposed as the central feared experience in dental phobia [29] and earlier findings in subjective data from our group [14]. As in earlier studies, DP showed no SCR differences compared to HC [13, 14, 30]. However, since pronounced responding towards auditory dental stimuli on a neural level was observed, this finding could indicate a dissociation between autonomic versus subjective and neural reactions as proposed earlier. The significant negative association between those brain regions involved in autonomic control such as the insula (as a trend) and the OFC [31, 32] and SC data may furthermore indicate inhibitory rather than excitatory regulation of autonomic outflow. This observation is also consistent with the often reported fainting response due to a relative vasovagal overshoot in BII phobics [33, 34]. Present findings could partly explain this observation in that subjective and neural elevations of fear may result in downregulation of autonomic reactions.

When comparing neural activation towards auditory versus visual information across groups, a pattern of increased activation in the ACC, insula, thalamus, and OFC was detected in DP. This result is also largely overlapping with core areas identified in animal specific phobia [5, 6]. Both ACC and insula were consistently found during phobic stimulus processing [13, 14, 35, 36], and hyperactivity in both structures recedes after successful cognitive-behavioral therapy [37]. Both insula and ACC have been related to threat evaluation processes [36] and anticipatory anxiety [38]. A recent study was also able to demonstrate a strong correlation between ACC and insula activation, albeit only in animal phobia [39]. Both have also been related to the neural response to disgust, being an emotion of particular importance in BII and dental phobia [40], but this seems to be the case for the insula to a greater extent [41]. Most notably, insula and ACC are also crucially involved in pain anticipation [42–44] and modulation of the experience of pain due to the perceived threat or anxiety level [45, 46]. In accordance with the corresponding pain ratings being significantly increased for auditory stimulus material on a subjective level, fear of pain seems to be relevant for the processing of drill sounds.

OFC activation in turn has rarely been investigated in specific phobia samples [47], but increased activation in this area seems to be relatively specific for DP compared to animal phobia [14]. Generally, activation in orbitofrontal and prefrontal gyri in DP has been related to processes of cognitive control and (re-)appraisal, possibly representing a more evaluation based fear response in DP than in animal phobia [9, 10, 14]. Such an evaluation-based response, being based on the sympathetically downregulating OFC rather than on the upregulating amygdala, is also well in line with the interpretation of diminished sympathetic responsiveness outlined above.

Neither the whole-brain nor the ROI approaches found any evidence for amygdala hyperactivation in this study. This lack of differential amygdala activation might be related to the general relevance of BII phobia stimuli applying to healthy subjects as well, as pointed out by Hermann et al. [11]. Additionally, besides contextual reasons, the block design used in this study could also have prevented the detection of amygdala activation [12, 14]. Future studies should combine auditory stimuli with an event-related fMRI task to further investigate whether the amygdala is recruited as well in rapid stimulus processing in DP.

Several limitations should be considered regarding the results of this study: DP were included on the basis of established clinical cut-offs, and future studies are needed to determine whether findings can be generalized to treatment-seeking patient samples. The size of the sample was relatively small, which might limit the ability to detect small scale effects. Additionally, the sample included DP with dental phobia only and DP with comorbid disorders. Due to the small size of both subgroups, an analysis of similarities and differences between these subgroups was omitted. Therefore, it is not clear whether the results of this study were significantly influenced by comorbidity. Furthermore, the study applied a block design that might prevent the finding of activity patterns in rapidly habituating structures.

## 5. Conclusion

This study aimed to investigate the impact of different stimulus modalities on subjective, autonomic, and neural threat processing in dental phobia. As such, it expands the literature on neural substrates of the disorder by showing evidence for the influence of stimulus modality. Auditory stimulation seems to be a more robust trigger of the neural network subserving threat processing in dental phobia, albeit subjective anxiety was elicited during both visual and auditory symptoms provocation. However, autonomic responding did not parallel neural activation but rather indicated a downregulation of autonomic outflow. Thus, when investigating the neural correlates of dental phobia, findings may partly depend on the modality of the used stimulus material. If replicated, these findings may help to understand and better distinguish the neural underpinnings and pathophysiology of these different specific phobia subtypes. Additionally, findings may also facilitate the improvement of clinical applications of phobic fear processing, for example, during exposure therapy, in the future.

## Figures and Tables

**Table 1:** Sample characteristics. Mean (SD) except where noted.

	HC (*n* = 13)	DP (*n* = 13)	chi^2^/*t* (df)	*P*
Sociodemographic characteristics				
Female sex (*n*. %)	9 (69.23)	9 (69.23)	—	—
Right-handed (*n*. %)	13 (100.0)	12 (92.31)	1.040	0.308
Unmarried (*n*. %)	12 (92.31)	11 (84.62)	0.377	0.539
Nonsmoker (*n*. %)	12 (92.31)	12 (92.31)	—	—
Age (years)	23.23 (3.19)	24.92 (2.25)	1.562	0.131

Clinical characteristics				
DFS^1^	25.77 (3.37)	79.54 (4.86)	32.788 (24)	<0.001
BDI	3.00 (2.89)	8.62 (9.59)	2.022 (24)	0.054
ASI	14.46 (7.82)	22.15 (12.08)	1.927 (24)	0.066
MQ	7.77 (5.97)	12.69 (6.05)	2.088 (24)	0.048

HC: healthy control group; DP: dental phobia group; DFS: Dental Fear Survey; BDI: Beck Depression Inventory-II; ASI: Anxiety Sensitivity Index; MQ: Mutilation Questionnaire; ^1^please note that questionnaire data relate to the date of screening that was used for study inclusion.

**Table 2:** Whole brain analysis on brain activation for group differences.

Group	Region	Side	Voxels	*F*	*P*	*x*	*y*	*z*
Stimulus: auditory, between-group: (DAA > DAN)								
DP > HC								
	ACC	L	954	5.91	<0.001	−4	32	22
	Calcarine sulcus	L	100	3.74	<0.001	−14	−62	18
	Hippocampus	L	78	3.94	<0.001	−20	−16	−8
	Insula	L	1858	5.34	<0.001	−30	12	−16
	Insula	R	515	4.48	<0.001	44	−12	10
	Postcentral gyrus	L	60	3.56	<0.001	−32	−42	54
	Precuneus	L	207	3.89	<0.001	−6	−58	46
	Inferior frontal gyrus (pars triangularis)	L	68	4.35	<0.001	−46	48	8
	Inferior frontal gyrus (pars triangularis)	L	76	3.98	<0.001	−50	14	26
	Thalamus	L	212	4.36	<0.001	−6	−8	4
HC > DP								
	MCC	L	107	4.86	<0.001	−12	−26	24
	MCC	R	88	4.29	<0.001	12	−12	30

Stimulus: visual, between-group: (DVA > DVN)								
DP > HC								
	Vermis	R	204	4.30	<0.001	8	−36	−34
HC > DP								
	No differential activation							

Stimulus: auditory versus visual, between-group: (DAA > DAN) > (DVA > DVN)								
DP > HC								
	Insula	L	326	4.93	<0.001	−32	14	−16
	Insula	R	165	4.44	<0.001	48	6	−6
	OFC	L	382	4.92	<0.001	−12	50	−6
	Precuneus	L	64	3.69	<0.001	−14	−58	40
HC > DP								
	No differential activation							

Stimulus: visual versus auditory, between-group: (DVA > DVN) > (DAA > DAN)								
DP > HC								
	Caudate nucleus	R	157	5.05	<0.001	28	−6	24
HC > DP								
	No differential activation							

HC: healthy control group; DP: dental phobia group; DAN: dental auditory neutral stimuli; DAA: dental auditory anxiety stimuli; DVN: dental visual neutral stimuli; DVA: dental visual anxiety stimuli; R: right side; L: left side; voxels: number of voxels per cluster; *x*, *y*, and *z*: MNI coordinates of peak voxel; ACC: anterior cingulate cortex; MCC: middle cingulate cortex; OFC: orbitofrontal cortex; analysis: minimum cluster size = 58; *P* < 0.001.

**Table 3:** Pearson's correlations between neural activation towards anxiety-inducing stimuli and DFS scores and phasic and tonic skin conductance reactivity in the dental phobia group (*n* = 13).

Brain areas (MNI coordinates)		DFS scores		CDA.SCR		CDA.tonic	
		*r*	*P* corr	r	*P* corr	r	P corr
Insula-L (auditory)	(−32, 14, −16)	−0.252	0.407	0.343	0.301	−0.538	0.088
Insula-L (visual)	(−32, 14, −16)	−0.109	0.722	−0.542	0.085	0.005	0.988
OFC-L (auditory)	(−12, 50, −6)	−0.315	0.294	0.480	0.135	−0.396	0.228
OFC-L (visual)	(−12, 50, −6)	−0.417	0.156	0.095	0.782	−0.642	0.033

DFS: Dental Fear Survey; CDA.SCR: phasic skin conductance reactivity; CDA.tonic: tonic skin conductance reactivity; OFC: orbitofrontal cortex; L: left side.
