# Corrigendum to “Fear Processing in Dental Phobia during Crossmodal Symptom Provocation: An fMRI Study”

**DOI:** 10.1155/2018/6497672

**Published:** 2018-11-06

**Authors:** Kevin Hilbert, Ricarda Evens, Nina Isabel Maslowski, Hans-Ulrich Wittchen, Ulrike Lueken

**Affiliations:** ^1^Institute of Clinical Psychology and Psychotherapy, Technische Universität Dresden, Chemnitzer Straße 46, 01187 Dresden, Germany; ^2^Neuroimaging Center, Technische Universität Dresden, Chemnitzer Straße 46a, 01187 Dresden, Germany

In the article titled “Fear Processing in Dental Phobia during Crossmodal Symptom Provocation: An fMRI Study” [1], an incorrect estimate of smoothness has been used in the Monte Carlo simulation used to determine the cluster-size based significance threshold [2]. As a result, the corresponding significance threshold for fMRI results was too conservative. Here we present our results table with the corrected significance threshold (minimum cluster size = 41).

Accordingly, in the method, the text reading “10000 iterations determined a minimum cluster size of 58 consecutive voxels” should be corrected to “10000 iterations determined a minimum cluster size of 41 consecutive voxels”.

In the fMRI results, the text reading “During visual stimulation, considerably less differential brain activation was found, with increased activation in the vermis in the DP being the only significant difference” should be corrected to “During visual stimulation, considerably less differential brain activation was found, increased activation in two parts of the cerebellum in the DP being the only significant differences” and “When finally comparing neural activation during auditory versus visual stimulation between groups, DP showed increased activation in the insula, OFC, and precuneus for auditory versus visual stimuli and in the caudate nucleus for visual versus auditory stimuli” should be corrected to “When finally comparing neural activation during auditory versus visual stimulation between groups, DP showed increased activation in the insula, OFC, superior temporal gyrus, thalamus, and precuneus for auditory versus visual stimuli and in the caudate nucleus for visual versus auditory stimuli”.

Finally, in the description of Figure 2, the text reading “analysis: minimum cluster size = 58; *∗* p < 0.05; *∗∗* p<0.01; *∗∗∗* p<0.001” should be corrected to “analysis: minimum cluster size = 41; *∗* p < 0.05; *∗∗* p<0.01; *∗∗∗* p<0.001”. The figure itself is still correct.

These additional results under the correct significance threshold do not invalidate the conclusions drawn in the original article, but rather substantiate the significant differences between crossmodal stimulus processing in dental phobia subjects and healthy controls originally reported and discussed. 

## Figures and Tables

**Table 2:** Whole brain analysis on brain activation for group differences.

Group/Phase	Region	Side	Voxels	F	p	x	y	z
*Stimulus: auditory, between-group: (DAA > DAN)*								
DP > HC								
	ACC	L	954	5.91	<0.001	-4	32	22
	Calcarine sulcus	L	100	3.74	<0.001	-14	-62	18
	Hippocampus	L	78	3.94	<0.001	-20	-16	-8
	Insula	L	1858	5.34	<0.001	-30	12	-16
	Insula	R	515	4.48	<0.001	44	-12	10
	Postcentral gyrus	L	60	3.56	<0.001	-32	-42	54
	Precuneus	L	207	3.89	<0.001	-6	-58	46
	Inferior frontal gyrus (pars triangularis)	L	68	4.35	<0.001	-46	48	8
	Inferior frontal gyrus (pars triangularis)	L	76	3.98	<0.001	-50	14	26
	Inferior frontal gyrus (pars opercularis)	R	46	3.75	<0.001	52	12	6
	Thalamus	L	212	4.36	<0.001	-6	-8	4
HC > DP								
	MCC	L	107	4.86	<0.001	-12	-26	24
	MCC	R	88	4.29	<0.001	12	-12	30
*Stimulus: visual, between-group: (DVA > DVN) *								
DP > HC								
	Vermis	R	204	4.30	<0.001	8	-36	-34
	Cerebellum	L	43	3.76	<0.001	-10	-44	-22
HC > DP								
	No differential activation							
*Stimulus: auditory versus visual, between-group: (DAA > DAN) > (DVA > DVN)*								
DP > HC								
	Insula	L	326	4.93	<0.001	-32	14	-16
	Insula	R	165	4.44	<0.001	48	6	-6
	OFC	L	382	4.92	<0.001	-12	50	-6
	Superior temporal gyrus	R	44	3.80	<0.001	60	-48	16
	Thalamus	L	47	3.72	<0.001	-2	-22	4
	Precuneus	L	64	3.69	<0.001	-14	-58	40
HC > DP								
	No differential activation							
*Stimulus: visual versus auditory, between-group: (DVA > DVN) > (DAA > DAN)*								
DP > HC								
	Caudate nucleus	R	157	5.05	<0.001	28	-6	24
HC > DP								
	No differential activation							

HC: healthy control group; DP: dental phobia group; DAN: dental auditory neutral stimuli; DAA: dental auditory anxiety stimuli; DVN: dental visual neutral stimuli; DVA: dental visual anxiety stimuli; R: right side; L: left side; Voxels: number of voxels per cluster; x, y, z: MNI coordinates of peak voxel; ACC: anterior cingulate cortex; MCC: middle cingulate cortex; OFC: orbitofrontal cortex; analysis: minimum cluster size = 41; p < 0.001.
